# Gene Polymorphisms in *FAS* (Rs3740286 and Rs4064) Are Involved in Endometriosis Development in Brazilian Women, but not those in *CASP8* (rs13416436 and rs2037815)

**DOI:** 10.1055/s-0038-1667183

**Published:** 2018-07-23

**Authors:** Cristina Wide Pissetti, Sarah Cristina Sato Vaz Tanaka, Andrezza Cristina Cancian Hortolani, Alessandra Bernadete Trovó de Marqui

**Affiliations:** 1 Department of Obstetrics and Ginecology, Universidade Federal da Paraíba, João Pessoa, PB, Brazil; 2 Department of Patology, Genetics and Evolution, Universidade Federal do Triângulo Mineiro, Uberaba, MG, Brazil

**Keywords:** endometriosis, apoptosis, genetic polymorphism, real-time polymerase chain reaction, genetic predisposition to disease, endometriose, apoptose, polimorfismo genético, reação em cadeia da polimerase em tempo real, predisposição genética para doença

## Abstract

**Objective** The present study aims to investigate the association between *caspase-8* (*CASP8*) (rs13416436 and rs2037815) and *Fas cell surface death receptor* (*FAS*) (rs3740286 and rs4064) polymorphisms with endometriosis in Brazilian women.

**Methods** In the present case-control study, 45 women with a diagnosis of endometriosis and 78 normal healthy women as a control group were included. The genotyping was determined by real-time polymerase chain reaction (PCR) with Taqman hydrolysis probes (Thermo Fisher Scientific, Darmstadt, Germany). Genotypic and allelic frequencies were analyzed using Chi-squared (χ^2^) test. In order to determine the inheritance models and haplotypes ,SNPStats (Institut Català d'Oncologia, Barcelona, Spain) was used. Levels of 5% (*p* = 0.05) were considered statistically significant.

**Results** No significant difference was observed in genotypic or allelic frequencies between control and endometriosis groups for rs13416436 and rs2037815 (*CASP8* gene). On the other hand, a significant difference between rs3740286 and rs4064 (*FAS* gene) was found. Regarding polymorphisms in the *FAS* gene, a statistically significant difference was found in co-dominant and dominant models. Only the haplotype containing the rs3740286A and rs4064G alleles in the *FAS* gene were statistically significant.

**Conclusion** The polymorphisms in the *CASP8* gene were not associated with endometriosis. The results indicate an association between *FAS* gene polymorphisms and the risk of developing endometriosis.

## Abstract (pt)

**Resumo:**

**Objetivo** Investigar a associação entre os polimorfismos dos genes *caspase-8* (*CASP8*) (rs13416436 e rs2037815) e *FAS* (rs3740286 e rs4064) em mulheres brasileiras com endometriose.

**Métodos** Trata-se de um estudo do tipo caso-controle, no qual foram incluídas 45 mulheres com diagnóstico de endometriose e 78 controles. A genotipagem das amostras foi determinada usando a reação em cadeia de polimerase em tempo real com sondas de hidrólise TaqMan (Thermo Fisher Scientific, Darmstadt, Germany). As frequências genotípicas e alélicas foram analisadas usando o teste do qui-quadrado. O SNPStats (Institut Català d'Oncologia, Barcelona, Espanha) foi usado para determinar os modelos de herança e os haplótipos. Os níveis de significância estatística considerados foram de 5% (*p* = 0,05).

**Resultados** Não foi observada diferença significativa nas frequências genotípicas ou alélicas entre os grupos de controle e de endometriose para os polimorfismos rs13416436 e rs2037815 (gene *CASP8*). Por outro lado, foi encontrada uma diferença significativa entre os polimorfismos rs3740286 e rs4064 (gene *FAS*). Em relação aos polimorfismos do gene *FAS*, foi encontrada uma diferença estatisticamente significativa nos modelos codominante e dominante. Apenas o haplótipo contendo os alelos rs3740286A e rs4064G no gene *FAS* foi estatisticamente significativo.

**Conclusão** Não há associação entre os polimorfismos do gene *CASP8* e endometriose. Entretanto, há associação entre os polimorfismos do gene *FAS* e o risco de desenvolver endometriose.

## Introduction

Endometriosis is a multifactorial disease that is characterized by the presence and growth of endometrial glands and stroma outside the uterus. It affects 10% of women in the reproductive age, and its main clinical manifestations include infertility, chronic pelvic pain, dysmenorrhea, and dyspareunia.^1^ ^2^

Previous studies showed that endometriosis-related symptoms significantly predict a negative impact on the daily life activities, work performance and social and marital life of the patients.^3^ ^4^ In addition, the results of a recent systematic literature review indicated that there is a significant economic burden associated with endometriosis, as observed by both direct and indirect costs. The direct costs included inpatient, outpatient, surgery, drug and other healthcare service costs. The indirect costs were related to absenteeism and presenteeism (loss of productivity at work).^5^

Laparoscopic exploration with histopathological examination is the gold standard for the diagnosis of endometriosis.^6^ During the surgery, lesion excision enables the histological confirmation of endometriosis based on the criteria of the American Society for Reproductive Medicine,^7^ in four stages: I (minimal disease), II (mild disease), III (moderate disease) and IV (severe disease).^7^ To prevent unnecessary surgery,^6^ the identification of a high-risk patient population for endometriosis with a complete clinical assessment should be performed, supported by a selective use of laboratory and imaging studies followed by surgery only on the high-risk population.

In women with endometriosis, the percentage of endometrial cells undergoing apoptosis is significantly decreased. On the other hand, the number of surviving cells is increased, and they still show physiological activity. The eutopic endometrium in women with endometriosis presents an increased expression of anti-apoptotic factor and a decreased expression of pro-apoptotic factors compared with the endometrium in healthy women. These differences could contribute to the survival of regurgitating endometrial cells into the peritoneal cavity and to the development of endometriosis.^8^ ^9^ The data in the literature indicate that apoptosis plays a critical role in the pathogenesis of endometriosis.^8^ ^9^ ^10^ Another study reviewed the role of apoptosis-associated molecules in the treatment of endometriosis with potential clinical applications in the future.^11^

Single nucleotide polymorphisms (SNPs) in apoptotic genes, such as those of the *Fas cell surface death receptor* (*FAS*) and the *caspase-8* (*CASP8*) genes, may be involved with the development of endometriosis.

Caspase-8 is a critical modulator of cell death, which initiates the apoptotic signaling via the extrinsic pathway, and plays a key role in the regulation of apoptosis.^12^

The FAS, also known as *TNFSF6*, *CD95*, or *APO-1*, is a cell surface receptor involved in the apoptotic signal transmission in many cell types that interacts with its natural *Fas ligand* known as FASL to initiate the death signal cascade that leads to apoptotic cell death.^13^ Although best characterized in terms of its apoptotic function, previous studies have identified several other cellular responses that include migration, invasion, inflammation, and proliferation.^14^ A recent study determined that stage III/IV endometriosis was associated with higher serum *CD95*/*FAS* and *hypoxia inducible factor 1 subunit alpha* (*HIF-1α*) levels, but not with *TEK receptor tyrosine kinase* (*Tie-2*) levels, compared with stage I/II endometriosis. These biomarkers may be useful for reproductive surgeons to improve the quality of counseling to women about the presence and the severity of endometriosis.^15^

Currently, genome-wide association studies (GWASs) have been very successful in identifying common genetic risk variants for several complex diseases. Genome-wide association studies have reported a significant association of endometriosis with chromosomes 2 and 10, which harbor *CASP8* and *FAS* genes respectively.^16^ ^17^ ^18^ ^19^ ^20^ ^21^

Therefore, the present study aims to investigate the association between *CASP8* (rs13416436 and rs2037815) and *FAS* (rs3740286 and rs4064) polymorphisms with endometriosis in Brazilian women.

## Methods

### Sample Characterization

This is a case-control study with 123 women treated in a public hospital in the interior of the state of Minas Gerais, Brazil. The women were divided into a control group (*n* = 78; 63.4%) and a case group (*n* = 45; 36.6%). The case group was characterized by the presence of endometriosis, and the control group, by the absence of it, both verified by laparoscopy or laparotomy. The inclusion factors were the surgical procedure that enabled the confirmation of the presence (patients) or absence (controls) of endometriosis, and a written informed consent form (WICF) for the participation in the present research. Women that have not met the aforementioned criteria were excluded. Among the women of the control group, the main reasons for surgical indication were tubal sterilization (24.4%), followed by chronic pelvic pain (7.7%) and infertility (6.4%). The total mean age was 39.1 (±9.6) years. In the control group, the mean age was 40.7 (±10.3) years, and in the endometriosis group, the mean age was 36.2 (±7.8) years.

Regarding the endometriosis staging, according to the American Society for Reproductive Medicine (ASRM),^7^ 6 women (13.4%) presented stage I disease, 1 (2.2%) presented stage II, 7 (15.5%) presented stage III, 1 (2.2%) presented stages III/IV, 12 (26.7%) presented stage IV, and in 18 women (40.0%) it was not possible to obtain this information.

All participants of the present study received an explanation about the present study and signed the WICF. The present research was approved by the Ethics Committee of Universidade Federal do Triângulo Mineiro (UFTM, in the Portuguese acronym) under protocol number 1628, and was conducted according to the principles described in the Declaration of Helsinki and in Resolution 466/2012 of the Brazilian National Health Council (CNS, in the Portuguese acronym). After signing the WICF, 10 mL of peripheral blood was drawn by venipuncture from the women who accepted participating in the present research.

### DNA Extraction and Genotyping

Using the salting-out protocol described by Miller et al,^22^ DNA was extracted from the peripheral blood samples collected in an ethylenediaminetetraacetic acid (EDTA) tube. The samples were then resuspended in Tris-EDTA (TE) 20:1, and the DNA integrity was verified in 1% agarose gel.

The four polymorphisms were genotyped via real-time polymerase chain reaction (PCR) with Taqman hydrolysis probes (Thermo Fisher Scientific, Darmstadt, Germany). The genotypes were determined by allelic discrimination. The chromosomal localizations of the polymorphisms rs13416436 and rs2037815 in the *CASP8* gene are Chr.2: 202099113 and Chr.2: 202101715 respectively. The chromosomal localizations of the polymorphism rs3740286 and rs4064 in the *FAS* gene are Chr.10: 90751340 and Chr.10: 90751380 respectively.

### Statistical Analysis

Hardy-Weinberg equilibrium (HWE) was evaluated by the Chi-squared (χ^2^)test, using Haploview 4.2 software (Broad Institute, Cambridge, MA, US), and the case group was in HWE. The statistical power was calculated using the G*Power 3.1.9.2 software (Heinrich-Heine University, Düsseldorf, Germany). A statistical power of 80% was obtained, with an effect size of 0.29 and an α level of significance of 0.05. Genotypic and allelic frequencies were analyzed using the Chi-squared test. Levels of 5% (*p* = 0.05) were considered statistically significant.

SNPStats (Institut Català d'Oncologia, Barcelona, Spain) was used to determine the haplotype and to perform the logistic regressed analysis for inheritance models, using codominant (major homozygotes versus heterozygotes versus minor homozygotes), dominant (major homozygotes versus heterozygotes plus minor homozygotes), and recessive (major homozygotes plus heterozygotes versus minor homozygotes). The risk estimates were expressed as the odds ratio (OR) with a 95% confidence interval (95%CI).

## Results

Concerning the polymorphisms studied, it was not possible to obtain the genotype of all of the participants of the present study, due to technical reasons – no amplification of the sample. For polymorphism *FAS* rs3740286 (A ˃ G) and *FAS* rs4064 (C ˃ G), 116 and 75 of the 123 samples were amplified respectively. For polymorphisms *CASP8* rs13416436 (A ˃ T) and *CASP8* rs2037815 (A ˃ G), the number of the samples amplified were 119 and 101 respectively.

For polymorphism *FAS* rs3740286 (A ˃ G), the association between the genotypes of the polymorphism and the development of endometriosis was observed (χ^2^ = 8.52; *p* = 0.014) (Table 1). To verify the association, the AG and GG genotypes were grouped in “G presence”, and the genotype AA, in “G absence.” It was observed that G presence was more frequent in the control group (χ^2^ = 8.51; *p* = 0.004). When the OR was calculated, the value obtained was 0.31 (95%CI = 0.14–0.69), suggesting that women with this allele have no presumed risk of developing endometriosis (Table 2).

**Table 1:** Frequency distribution of the genotypes of polymorphisms *FAS* rs3740286 (A ˃ G), *FAS* rs4064 (C ˃ G), *CASP8* rs13416436 (A ˃ T) and *CASP8* rs2037815 (A ˃ G), in women with endometriosis and in the control group

Diagnosis							
	Control group		Endometriosis		Total		
Polymorphism/genotype	*n*	%	*n*	%	*n*	%	
rs3740286(A ˃ G)							
AA	26	51.0	25	49.0	51	100	*p* = 0.014
AG	36	76.6	11	23.4	47	100	
GG	14	77.8	04	22.2	18	100	
Total	76	65.5	40	34.5	116	100	
rs4064 (C ˃ G)							
CC	26	76.5	08	23.5	34	100	*p* = 0.039
GC	17	56.7	13	43.3	30	100	
GG	04	36.4	07	63.6	11	100	
Total	47	62.7	28	37.3	75	100	
rs13416436 (A > T)							
AA	02	100	00	0.00	02	100	*p* = 0.42
AT	15	71.4	06	28.6	21	100	
TT	60	62.5	36	37.5	96	100	
Total	77	64.7	42	35.3	119	100	
rs2037815 (A > G)							
AA	21	60.0	14	40.0	35	100	*p* = 0.39
AG	33	64.7	18	35.3	51	100	
GG	12	80.0	03	20.0	15	100	
Total	66	65.3	35	34.7	101	100	

**Table 2:** Frequency distribution of the presence of the G allele of the polymorphism *FAS* rs3740286 and *FAS* rs4064, presence of an allele of the polymorphism *CASP8* rs13416436 and presence of the G allele of the polymorphism *CASP8* rs2037815, in women with endometriosis and in the control group

Diagnosis							
	Control group		Endometriosis		Total		
G presence rs3740286	*n*	%	*n*	%	*n*	%	
No	26	51.0	25	49.0	51	100	*p* = 0.004
Yes	50	76.9	15	23.1	65	100	OR = 0.31
Total	76	65.5	40	34.5	116	100	
G presence rs4064							
No	26	76.5	08	23.5	34	100	*p* = 0.024
Yes	21	51.2	20	48.8	41	100	OR = 3.09
Total	47	62.7	28	37.3	75	100	
A presence rs13416436							
No	60	62.5	36	37.5	96	100	*p* = 0.30
Yes	17	73.9	06	26.1	23	100	OR = 0.59
Total	77	64.7	42	35.3	119	100	
G presence rs2037815							
No	21	60.0	14	40.0	35	100	*p* = 0.41
Yes	45	68.2	21	31.8	66	100	OR = 0.7
Total	66	65.3	35	34.7	101	100	

Abbreviation: OR, odds ratio.

Regarding the polymorphism *FAS* rs4064 (C ˃ G), there was an association between the genotypes of the polymorphism studied and the development of endometriosis (χ^2^ = 6.48; *p* = 0.039) (Table 1). To verify the association, the GC and GG genotypes were grouped in “G presence”, and the CC genotype was grouped in “G absence.” The absence of the G allele was more frequent in the control group (χ^2^ = 5.06; *p* = 0.024). When the OR was calculated, the value obtained was 3.09 (95%CI = 1.14–8.43), suggesting that the absence of the G allele confers a protection 3 times higher against the development of endometriosis in comparison to women who present this allele (Table 2).

However, when the polymorphisms in the *CASP8* gene were assessed, no association between the polymorphisms studied (rs13416436 and rs2037815) and the development of endometriosis was found. For the polymorphism *CASP8* rs13416436 (A ˃ T), no association between the genotypes of the polymorphism studied and the development of endometriosis was found (χ^2^ = 1.71; *p* = 0.42) (Table 1). The AA and AT genotypes were grouped in “A presence”, and the TT genotype was grouped in “A absence” to assess if there was an association between the presence of the A allele and the susceptibility to the development of endometriosis. However, no statistically significant differences were found (χ^2^ = 1.06; *p* = 0.30; OR = 0.59; 95%CI = 0.21–1.63) (Table 2).

Regarding the polymorphism *CASP8* rs2037815 (A ˃ G), there was no association between the genotypes of the polymorphism studied and the development of endometriosis (χ^2^ = 1.87; *p* = 0.39) (Table 1). The AG and GG genotypes were grouped in “G presence”, and the AA genotype was grouped in “G absence” to evaluate if there was an association between the presence of the G allele and the susceptibility to develop endometriosis. However, no statistically significant difference was found (χ^2^ = 0.68; *p* = 0.41; OR = 0.7; 95%CI = 0.3–1.64) (Table 2).

Regarding the polymorphisms rs13416436 (A > T) and rs2037815 (A ˃ G) in the *CASP8* gene, no statistically significant difference was found in any of the genetic models analyzed (codominant: *p* = 0.34 and *p* = 0.34; dominant: *p* = 0.26 and *p* = 0.25; recessive: *p* = 0.24 and *p* = 0.23 respectively). Regarding the polymorphisms rs3740286 (A > G) and rs4064 (G > C) in the *FAS* gene, a statistically significant difference was found in the codominant and dominant models (*p* = 0.02, *p* = 0.04; and *p* = 0.006, *p* = 0.01 respectively) (Table 3).

**Table 3:** *FAS* and *CASP8* gene polymorphism analysis in different genetic models

Gene/SNP	Model	OR (95%CI)	*p*-value
*FAS* rs3740286	Codominant (AA x AG x GG)	1.00	
		3.03 (1.24–7.41)	0.02
		3.24 (0.92–11.35)	
	Dominant (AA x AG + GG)	1.00	
		3.09 (1.36–6.99)	0.006
	Recessive (AA + AG x GG)	1.00	
		1.99 (0.60–6.59)	0.24
*FAS* rs4064	Codominant (GG x CG x CC)	1.00	
		0.35 (0.11–1.06)	0.04
		0.20 (0.04–0.88)	
	Dominant (CC x CG + GG)	1.00	
		0.30 (0.10–0.84)	0.01
	Recessive (CC + CG x GG)	1.00	
		0.33 (0.08–1.29)	0.11
*CASP8* rs13416436	Codominant (TT x AT x AA)	1.00	
		1.64 (0.56–4.76)	0.34
		−	
	Dominant (TT x AT + AA)	1.00	
		1.80 (0.63–5.16)	0.26
	Recessive (TT + AT x AA)	1.00	
		−	0.24
*CASP8* rs2037815	Codominant (AA x AG x GG)	1.00	
		1.49 (0.59–3.77)	0.34
		2.80 (0.64–12.20)	
	Dominant (AA x AG + GG)	1.00	
		1.49 (0.59–3.77)	0.25
	Recessive (AA + AG x GG)	1.00	
		2.21 (0.56–8.66)	0.23

Abbreviations: 95%CI, 95% confidence interval; OR, odds ratio.

The prevalence of haplotypes in the polymorphisms rs13416436 (A > T) and rs2037815 (A ˃ G) in the *CASP8* gene, and the rs3740286 (A > G) and rs4064 (G > C) in the *FAS* gene is shown in Table 4. Only the haplotype containing the rs3740286A and rs4064G alleles in the *FAS* gene was statistically significant (OR = 0.33; 95%CI = 0.15–0.72; *p* = 0.0062).

**Table 4:** Haplotype analysis between rs13416436 (A > T) and rs2037815 (A > G) of the *CASP8* gene and rs3740286 (A > G) and rs4064 (G > C) in the *FAS* gene on the risk of developing endometriosis

Gene	Haplotype	ED	Control	OR (95%CI)	*p-value*
*CASP8*	T-A	0.6494	0.4459	1.00	—
	T-G	0.2824	0.4308	1.74 (0.89 - 3.39)	0.11
	A-A	0	0.1234	2.10 (0.77 - 5.77)	0.15
	A-G	0.0682	0		
*FAS*	G-C	0.2111	0.4048	1.00	—
	A-G	0.4489	0.2558	0.33 (0.15–0.72)	0.0062
	A-C	0.3139	0.3231	0.66 (0.31–1.39)	0.27
	G-G	0.0262	0.0162	1.17 (0.07–20.52)	0.92

Abbreviations: 95% CI, 95% confidence interval; ED, endometriosis; OR, odds ratio.

## Discussion

According to the data in the literature, apoptosis plays an important role in the development of endometriosis.^8^ ^9^ ^10^ A recent review aimed to shed light on the role of the apoptosis pathways in the modulation of the fine-regulated peritoneal microenvironment during endometriosis.^23^ Considering the large amount of evidence retrieved from in vitro as well as in vivo models, the reduced apoptosis of endometriotic cells together with the increased apoptosis of peritoneal fluid mononuclear cells may address the peritoneal homeostasis to a permissive environment for the progression of the disease.^23^

Endometriosis is a gynecologic condition characterized by the growth of endometrial tissue outside the uterus. Therefore, genes that regulate the growth and the reproduction of endometrial cells and genes that aid the survival of cells and eliminate apoptosis are activated.^8^ ^9^ ^11^ This is the reason why, in connection with endometriosis, we have focused on the analysis of polymorphisms associated with apoptosis. It is important to search for biomarkers that could be useful to determine the predisposition and/or the prognosis.

In the present study, we have hypothesized that genetic factors are involved in the etiology of endometriosis; therefore, our aim was to evaluate the genetic predisposition to the development of endometriosis regarding the presence of four polymorphisms: rs13416436, rs2037815, rs3740286 and rs4064.

The polymorphisms rs13416436 and rs2037815 are characterized by an A/T and A/G single-nucleotide variation respectively, on human chromosome 2, while SNPs rs3740286 and rs4064 are located on chromosome 10 and correspond to A/G and C/G alterations respectively. The present study was the first to analyze the possible associations of these polymorphisms with endometriosis in a Brazilian sample population.

The present study indicated the absence of association between polymorphisms in the *CASP8* gene and the risk of developing endometriosis. Our results are in line with a previous study conducted by our group.^24^ There are no studies on the polymorphisms rs13416436 and rs2037815 of the *CASP8* gene regarding endometriosis. Only two studies analyzed these polymorphisms in preeclampsia and multiple sclerosis (MS).^24^ ^25^ Orlando et al^24^ showed the absence of association between rs13416436 and rs2037815 with the development of preeclampsia. However, for SNP rs2037815, GG homozygosity was associated with cases of primary progressive MS when compared with cases of relapse-onset MS and controls.^25^

Previous studies using genome-wide association (GWA) analysis have identified susceptibility genes for endometriosis in chromosome 2.^17^ ^18^ ^20^ ^26^ Adachi et al^17^ showed that four of the top five SNPs were in and around interleukin 1α (IL1A) at 2q13, which might be a functional candidate gene for endometriosis. Another GWA meta-analysis in 4,604 cases of endometriosis and 9,393 controls identified 7 SNPs associated with endometriosis, 2 of them in chromosome 2 (rs13394619*–*2p25.1 and rs4141819–2p14).^18^ Sundqvist et al^20^ observed a weak association with endometriosis (all stages) for rs1250248 in the 2q35 locus (*p* = 0.049). A recent meta-analysis showed a remarkable consistency in endometriosis GWA results across studies, with little evidence of population-based heterogeneity.^26^ It also recommended functional studies in relevant tissues to understand the effect of the variants on downstream biological pathways.^26^ An interesting finding common to these three researches^17^ ^18^ ^20^ was the identification of the polymorphisms associated with endometriosis in chromosome 2, in which the *CASP8* gene, investigated in the present study, is located. However, despite the evidence of the participation of this chromosome in the etiology of endometriosis, the present study has not associated the polymorphism of the *CASP8* gene with the disease.

Regarding the two *FAS* SNPs, our results suggest a significant effect on the susceptibility to endometriosis. Only one study evaluated the three polymorphisms located within the *FAS* (-1377 G > A and -670 A > G) and *FASL* (-843 C > T) genes as susceptibility factors for endometriosis.^27^ The results indicated that the variants analyzed are not involved in the pathogenesis of the disease in the sample . The authors suggest that a complete genetic analysis of the genes involved in the intricate regulatory system of the apoptosis may lead to the identification of susceptibility factors for the disease and a better understanding of its etiology.^27^ In spite of showing an absence of association between *FAS* polymorphisms and endometriosis, Fernández et al^27^ reported that this does not allow us to completely exclude these genes as potential candidates for the disease. Our results corroborate this finding.

Although we have identified an association of polymorphisms in chromosome 10 with susceptibility to endometriosis, another study showed that polymorphisms in the *cytochrome P450 family 17 subfamily A member 1* (*CYP17A1*) and *interferon-induced protein with tetratricopeptide repeats 1*(*IFIT1*) genes in chromosome 10 did not contribute to the risk of endometriosis in the Australian population.^28^ A systematic literature review conducted in 2008 showed that: 1) there is evidence of genetic linkage to chromosomes 7 and 10; 2) genetic variants in 76 genes were associated with endometriosis; and 3) GWAs are recommended to locate the genetic variants that contribute to a range of common diseases.^29^

The present study is limited due to the small sample size, which decreased our ability to solidify statistic associations. Despite the small sample size, the post hoc statistical power was 80%. Since the endometriosis patients recruited in our study are all Brazilian, the association between these four polymorphisms and other populations should also be investigated. In summary, further studies with different ethnic populations and with a larger sample could help to confirm the true significance of the association between these polymorphisms and the risk of endometriosis. Another limitation of the present study was the absence or lack of scientific works on these polymorphisms in endometriosis and other biological conditions, which has made the data generalization and comparison difficult.

A strong point of the present study is that all women who participated (cases and controls) were surgically evaluated to test for endometriosis. In addition, the present work is the first study to focus on the possible contribution of apoptosis-related gene polymorphisms to the development of endometriosis.

## Conclusion

The polymorphisms in the *CASP8* gene are not associated with endometriosis. The results indicate a positive association between the rs3740286 and rs4064 of the *FAS* gene and the risk of developing endometriosis. Therefore, further studies on the functional relevance of the *CASP8* and *FAS* polymorphisms are required to confirm our observations.
